# Bleeding in the Chill of Intermittents

**Published:** 1836

**Authors:** A. G. Henry

**Affiliations:** Springfield, Ill.


### Art. V.—Bleeding in the Chill of Intermittents.

*Cases displaying the efficacy of Blood-letting in the cold stage of Intermittent Fever.* In a letter to the Editor.

Dear Sir,—The following cases and remarks were published in the "Register and Library of Medical and Chirurgical Science," in 1834; and if I mistake not, are the first cases that have been published in the United States, (if we except the case alluded to by Dr. Eberle in his Practice,) confirming the propriety of blood-letting in the cold stage of intermittents, as recommended by Doctor Mackintosh of Edinburgh. Subsequent experience has fully confirmed my views as then expressed. I feel anxious to see the attention of the profession in the West drawn to the subject; believing as I do, that a fair trial of the practice, is all that is necessary to inspire the fullest confidence in its efficacy. I notice that the practice has recently been adopted in India, by Doctor Campbell, with signal success, and that Mr. Twining, (whose unbounded experience and sound judgment ought to entitle the practice to the favorable consideration of the profession,) has given it his sanction.

Case 1st. Sales, aged about 45, and weighing upwards of 290, of strong constitution, having enjoyed uninterrupted good health for many years, was seized with severe rigors on the 6th of August, 1834, which continued for four or five hours, accompanied with great difficulty of breathing; and so great were his sufferings that serious apprehensions were entertained by his physician of a fatal termination in the next paroxysm. The hot stage was usually severe, and lasted six or seven hours. I was requested to see him the next day. I did so, and found him beginning to feel chilly and breathe laboriously, exhibiting marks of great suffering. I used the ordinary remedies; but the symptoms becoming every moment more urgent, and believing that unless speedy relief was obtained, death would be the consequence, as a last resort I determined on trying Doctor Mackintosh's plan of blood-letting. Accordingly I opened a vein in the arm, and with much difficulty succeeded in getting 12 or 13 oz. blood. So soon as the blood commenced running freely, the pulse, from being almost imperceptible, became fuller and perfectly distinct; the rigors ceased, and every symptom which a moment before had threatened a speedy dissolution, was greatly relieved. This continued for eight or ten minutes, when the symptoms returned in all their former violence. I reopened the vein and took 15 or 16 oz. more, which gave permanent relief to all the distressing symptoms. The skin soon became moist; and no hot or sweating stage followed. I directed 10 grains of quinine to be taken during the night; he recovered his usual health in three or four days, and has had no recurrence of the disease since.

The astonishing effects of bleeding in this case, inspired the fullest confidence in Dr. Mackintosh's mode of treatment, and induced me to resort to it in the following cases without any dread of consequences.

Case 2d. Humphries West, laborer, was taken with a tertian ague on the 18th day of August, 1834. His rigors were strong and continued for an hour and a half, and accompanied with distressing retching and vomiting. I saw him for the first time in the last stage of the second paroxysm. I made arrangements to be in time to anticipate the next regular paroxysm, which I accordingly did. I waited until the cold stage became perfectly formed, and he had begun to shake violently, for the purpose of demonstrating more fully the power of the remedy. I then opened a vein, and with less difficulty than I anticipated, succeeded in getting 6 or 8 oz. of blood, when the shaking ceased entirely, the skin became moist, and he expressed himself as feeling entirely relieved. No hot or sweating stage followed. Eight grs. of quinine, combined with four of piperine were directed to be taken in the course of the next day ; no paroxysm followed.

Case 3d. Matthew Crowder, laborer, aged 35 and of full habit, was taken on the 28th of August, 1834, with a quotidian ague. I saw him an hour before the third expected paroxysm. His general appearance was indicative of his having suffered severely during the previous paroxysms, the rigors having continued near two hours, accompanied with a distressing sense of suffocation, and the hot fit lasting within three or four hours of the next paroxysm. I waited, as before, until the cold stage had become fully formed, when I opened a vein; but in consequence of the operation having been badly performed, I did not succeed in getting more than three or four oz. of blood; this quantity evidently relieved the symptoms for a few moments, when I opened a vein in the other arm, and by the aid of frictions soon got the blood to flowing freely, when the rigors suddenly ceased. I let the blood flow to the extent of 15 or 16 oz. when the pulse rose, and became full and soft. He now declared he felt better than he had done at any time since his first attack. No hot fit followed, he continued free from any unpleasant symptoms, and with the assistance of a few ague pills, he rapidly recovered.

Case 4. George Kingsley, brick mason, aged 25, was attacked with a tertian ague on the 6th of December, 1834, from exposure to wet and cold. I saw him in the hot stage of the first paroxysm. His fever ran high, and lasted eight or nine hours, complaining of severe pain in the head and back. I directed a mild cathartic of rhei to be taken that night, and promised to see him at the commencement of next fit; found him shaking violently; no pulse perceptible at the wrist; ghastly expression of countenance, and very difficult breathing. I opened a vein in the arm, being guided by the old cicatrix, (not being able to raise a vein sufficiently to distinguish it,) and by persevering with frictions, I succeeded in half an hour in getting the blood to flow freely in a full stream, when the pulse became full and distinct, the shaking ceased entirely, skin became moist, and he expressed himself as feeling better than he did in the sweating stage of the previous paroxysm. No hot or sweating stage followed, and with the aid of a few grains of quinine and piperine taken the next day, went to his business the day after, feelıng perfectly well.

Several other cases where the cold stage was comparatively light, have been treated on this plan with the same happy results, under my direction, or by myself. One case in particular, which was treated by a student of mine, is worthy of notice. A brother of his, aged 16, had labored under tertian ague for two months without interruption, although large quantities of quinine had been used. A vein was opened on the first appearance of chill (which I regard as important, as you thereby prevent the cold stage almost entirely, and get blood with greater ease;) the symptoms were all relieved; no fever followed that day, and no recurrence of the disease has since taken place.

I will only add in conclusion, that I do not entertain a doubt of the correctnsss of Doctor Mackintosh's views in relation to the pathology and treatment of Intermittent Fever; and that I am happy in having it in my power to offer in confirmation the foregoing cases. I regard the employment of blood-letting in the cold stage of intermittents, as the most important improvement in the practice of modern times; and notwithstanding the overbearing influence of authority, I believe the time is not far distant when it will become the established practice of the profession.

I have extended the practice to the treatment of remittent fevers; and no longer, as formerly, wait until reaction is at its height before I resort to the lancet. I have repeatedly this season bled in our bilious remittent fevers, when the feet and hands were cold, pulse small and feeble, with the most happy results. One case in particular, where the attack was unusually violent, and threatening a fatal termination (two cases having terminated fatally in the neighborhood under similar circumstances in the course of three days,) I bled to the extent of forty oz. in all, at three different times in the course of two hours, with relief to all the symptoms at each bleeding. The pulse rose under the last operation; the reaction which followed was trifling compared with what it is in ordinary cases of remittent fever. The subsequent practice consisted of five grains of calomel at night, followed with a seidlitz in the morning for four successive days, when the patient was dismissed cured.

Respectfully yours,

A. G. Henry.

Springfield, Ill., October, 6th, 1836.

